# Extensive GJD2 Expression in the Song Motor Pathway Reveals the Extent of Electrical Synapses in the Songbird Brain

**DOI:** 10.3390/biology10111099

**Published:** 2021-10-25

**Authors:** Pepe Alcami, Santhosh Totagera, Nina Sohnius-Wilhelmi, Stefan Leitner, Benedikt Grothe, Carolina Frankl-Vilches, Manfred Gahr

**Affiliations:** 1 Department of Behavioural Neurobiology, Max Planck Institute for Ornithology, Eberhard-Gwinner-Straße, 82319 Starnberg, Germany; s.totagera@campus.lmu.de (S.T.); ninasohnius@web.de (N.S.-W.); leitner@orn.mpg.de (S.L.); frankl@orn.mpg.de (C.F.-V.); gahr@orn.mpg.de (M.G.); 2 Division of Neurobiology, Faculty of Biology, Ludwig-Maximilians-Universität München, Grosshaderner Strasse 2, D-82152 Planegg-Martinsried, Germany; grothe@lmu.de

**Keywords:** electrical synapse, GJD2, connexin 36, birdsong, song nuclei, premotor networks, HVC, RA, nidopallium, arcopallium

## Abstract

**Simple Summary:**

Electrical synapses are ubiquitous in nervous systems, where they coordinate the activity of neural networks in time. However, their role in the execution and learning of complex behaviors remains unknown. Electrical synapses have remained unexplored in the songbird brain, which provides a model to link the production and learning of a complex behavior to the synaptic structure of defined neural circuits. Here, we show that GJD2 mRNA, coding for the major channel-forming electrical synapse protein connexin 36 (Cx36), is extensively expressed in the two nuclei that control song production, HVC and RA and their embedding regions. Our in situ hybridizations, together with the analysis of published transcriptomics data, demonstrate that electrical synapses are a general and widespread feature of song premotor nuclei in songbirds, where they show brain region-specific, cell type-specific expression patterns, dynamic during neuronal differentiation. We propose songbirds as a suitable model to investigate the contribution of the major vertebrate electrical synapse protein, Cx36, to the production and learning of motor skills in vertebrates.

**Abstract:**

Birdsong is a precisely timed animal behavior. The connectivity of song premotor neural networks has been proposed to underlie the temporal patterns of neuronal activity that control vocal muscle movements during singing. Although the connectivity of premotor nuclei via chemical synapses has been characterized, electrical synapses and their molecular identity remain unexplored. We show with in situ hybridizations that GJD2 mRNA, coding for the major channel-forming electrical synapse protein in mammals, connexin 36, is expressed in the two nuclei that control song production, HVC and RA from canaries and zebra finches. In canaries’ HVC, GJD2 mRNA is extensively expressed in GABAergic and only a fraction of glutamatergic cells. By contrast, in RA, GJD2 mRNA expression is widespread in glutamatergic and GABAergic neurons. Remarkably, GJD2 expression is similar in song nuclei and their respective embedding brain regions, revealing the widespread expression of GJD2 in the avian brain. Inspection of a single-cell sequencing database from zebra and Bengalese finches generalizes the distributions of electrical synapses across cell types and song nuclei that we found in HVC and RA from canaries, reveals a differential GJD2 mRNA expression in HVC glutamatergic subtypes and its transient increase along the neurogenic lineage. We propose that songbirds are a suitable model to investigate the contribution of electrical synapses to motor skill learning and production.

## 1. Introduction

Electrical synapses play a major role in coordinating the activity of neural networks in both invertebrate and vertebrate nervous systems [1,2,3]. They can lead to variable effects on the collective patterns of activity of electrically coupled networks. These include the regulation of action potential firing synchronization [4,5,6], the excitation or the inhibition of coupled cells, the detection of coincident and sequential inputs in coupled neural networks [1,5,6,7] and the redefinition of neural compartments [1,8]. In most mammalian brain regions, both electrical and chemical synapses have been shown to coexist and, together, contribute to the excitability and dynamics of neural networks [4,7,9,10]. Electrical synapses are formed by specialized channel-forming proteins contributed by coupled cells. Once channels from two adjacent cells dock to each other, they link the cytosol of coupled cells, creating intercellular pathways that allow ion flow and, thereby, electrically couple cells. In vertebrates, gap junction channel-forming proteins belong to the connexin (Cx) family. Cx36, encoded by the GJD2 gene, has been shown to be the substrate of the large majority of electrical synapses in mammals [11]. Despite GJD2 being found in the avian genome and expressed in the developing nervous system and the retina [12,13,14,15], GJD2 expression in the avian brain has not been investigated yet.

The songbird brain contains nuclei specialized in the control of the timing and spectral properties of birdsong, a complex behavior encoded in the temporal dynamics of premotor network activity [16,17]. Two premotor nuclei in the songbird brain play a major role in controlling the properties of birdsong: HVC (proper name) and the RA (the robust nucleus of the arcopallium). Both brain regions are well known for containing networks that reliably encode the timing and spectral properties of songs produced by songbirds [16,18,19]. Although a role for chemical synapses has been proposed in the coordination of neural activity in both nuclei [20,21,22], the existence of electrical synapses and their contribution to the temporal activation of HVC and RA neurons remains unexplored. Attempts to model HVC dynamics indeed rely on chemical synapses between HVC principal cells, which, however, appear to be sparse and whose contribution to generating sequences of neural activity in HVC remains controversial [23]. Chemical inhibitory synapses have additionally been proposed to contribute to the generation of firing sequences [22]. Finally, chemical synapses within nucleus RA have been shown to contribute to the coding of song features, without affecting the temporal structure of songs [21].

In this article, we demonstrate with in situ hybridizations the expression of GJD2 mRNA in both HVC and RA of two songbird model species in the behavioral neurosciences: the canary (*Serinus canaria*) and the zebra finch (*Taeniopygia guttata*), and we quantify at the cellular resolution the expression of GJD2 by glutamatergic and GABAergic neurons in both nuclei from canaries and their embedding regions. We additionally confirm the relative expression of GJD2 in excitatory and inhibitory cells in HVC and RA from previously published RNA sequencing in the zebra finch and the Bengalese finch and analyze GJD2 expression in HVC excitatory subtypes and in the neurogenic lineage.

## 2. Materials and Methods

### 2.1. Animals

Nine adult male canaries (*Serinus canaria*), 10 to 45 months old, and one 3 month-old adult male zebra finch from an outside aviary were collected year long (April 2017–January 2018) from aviaries at the Max Planck Institute for Ornithology in Seewiesen. Birds were housed together with females throughout the year and kept under natural light conditions in an aviary with an outdoor compartment (416 × 242 × 302 cm) and an inside shelter (403 × 301 × 200 cm) throughout the study period. The natural photoperiod varied from 16:8 L/D in summer to 10:14 L/D in winter. Birds were euthanized by an overdose of isoflurane, and their brains were snap-frozen on dry ice. Samples were taken at different times of the year in order to avoid any seasonal bias.

### 2.2. Fluorescence in Situ Hybridizations

Brains were sectioned sagittally into 3 series of 20-μm sections with a cryostat (Jung CM3000 Leica, Wetzlar, Germany) and mounted on Superfrost slides (Thermofisher Scientific, Braunschweig, Germany). Slices were fixed with 4% paraformaldehyde (PFA) solution at 4 °C for 15 min and dehydrated with 50%, 70% and 100% ethanol solution for 5 min at room temperature. Samples were pretreated with RNAscope^®^ Protease IV (ACDBio Pretreatment Catalog Number: 322336) for 30 min at room temperature, followed by two washing steps in MilliQ water. We then performed the RNAscope^®^ Fluorescent Multiplex Assay. Tissues were hybridized with GJD2, VGLUT2 and GAD2 probes designed by ACDBio. The following gene sequences were targeted by probes: RNAscope^®^ Probe, Tgu-SLC17A6-C3; RNAscope^®^ Probe, Tgu-GAD2-C2; and the Tgu-GJD2 C1 targeting 462-1440 of XM_002199609.2 (covering nucleotides 655-1623 from XM_030274566 based on the new version of the zebra finch genome annotation, release: 106, Assembly ID: bTaeGut1.4.pri). Probes were incubated in a humidified chamber at 40 °C for 2 h. Then, amplification steps were performed using RNAscope^®^ Fluorescent multiplex reagents (ACDBio Manual ID: 320293) Amp1, Amp2, Amp3 and Amp 4A ltB serially for 30 min, 15 min, 30 min, and 15 min, respectively, at 40 °C in a HybEZ™ Hybridization System. Finally, sections were stained with DAPI for several seconds and mounted in Vectashield mounting medium (Vector Laboratories, VECTH-1000).

### 2.3. Confocal Microscopy

Confocal optical sections were acquired with a Leica TCS SP5-2 confocal laser-scanning microscope (Leica Microsystems, Mannheim, Germany) equipped with objectives HCX PL APO CS 20×/numerical aperture 0.7 and 63× HCX PL APO lambda blue/numerical aperture 1.4. Fluorochromes were visualized with excitation wavelengths of 405 nm for DAPI, 488 nm for Alexa Fluor-488, 561 nm for Atto 550 and 633 nm for Atto-647.

### 2.4. Analysis

Images were analyzed using ImageJ 1.53a software (U.S. National Institutes of Health, Bethesda, MD, USA; https://imagej.nih.gov/ij/ accessed on 4 May 2020). Quantification of GJD2 puncta was performed manually from single confocal planes from one plane or two adjacent planes per animal at 63× objective magnification for the brain regions HVC, RA, nidopallium and arcopallium. VGLUT2 and GAD2 puncta were used to delineate the boundaries of glutamatergic and GABAergic cell types, respectively. 4′,6-Diamidino-2-phenylindole (DAPI) nuclear counterstaining was additionally used as a criterion to delineate cells and categorize the drawn regions of interest (ROIs) as single neurons or, instead, as clusters of neurons.

We only considered GAD2-positive and VGLUT2-positive cells imaged close to their equatorial plane in order not to bias for low densities of puncta by considering small cell areas. We, therefore, defined GAD2- and VGLUT2-positive cells as cells for which a DAPI-stained cell nucleus was observed and GAD2 or VGLUT2 puncta were found around the nucleus. GAD2 and VGLUT2 transcripts consisted in all cases of several puncta, and no minimal number of puncta was taken into account to consider a cell positive for VGLUT2 or GAD2. The average number of excitatory and inhibitory neurons per animal and region was 27.1 (range: 13–57) and 17.9 (range: 5–30), respectively. Once cells were identified as VGLUT2 positive or GAD2 positive, their contour was drawn. All GJD2 puncta detected inside this contour were considered positive, applying no threshold on the minimal puncta number or density to consider a cell positive for GJD2. GJD2 puncta were identified manually based on circularity criteria (elliptic shape), signal intensity (only high-intensity puncta were considered to be in the plane) and size (large signals beyond the stereotypical punctum size were excluded and the smallest punctum considered for HVC quantifications consisted of 23 pixels, pixel size = 60 nm). The number of puncta was divided by the area of respective ROIs to calculate the density of GJD2 expression. Randomly placed ROIs in regions of the image containing no DAPI staining and no staining for any of the markers of excitatory and inhibitory populations, VGLUT2 and GAD2, were used to quantify background GJD2 expression (these ROIs are labeled ‘random’ in Figures 4–7). For ’random’ ROIs, the average GJD2 mRNA density per brain region in a given animal was quantified from densities in the ROIs expressing at least one GJD2 punctum, similar to the procedure to quantify GJD2 density on GAD2 or VGLUT2 mRNA-positive ROIs. However, in few animals and regions, none of the random ROIs expressed GJD2. In these cases, we used all random ROIs, irrespective of the presence of GJD2 puncta, to compute the average GJD2 density, which was null. Figures were generated with ImageJ and Igor Pro software.

### 2.5. Cellular Transcriptomics Dataset

We used the HVC_RA_X_normalized.csv dataset to analyze the expression of GJD2 in zebra and Bengalese finches: https://cloud.biohpc.swmed.edu/index.php/s/nLicEtkmjGGmRF8?path=%2F (accessed on 2 July 2021).

### 2.6. Statistics

The paired two-tailed Wilcoxon signed-rank test, the two-tailed Wilcoxon–Mann–Whitney two-sample rank test and Fisher’s exact test were performed as specified in the manuscript with Igor Pro software (wavemetrics, OR, USA; www.wavemetrics.com, version 8.02, accessed on 10 September 2018) and GraphPad Prism 9 (San Diego, CA, USA; https://www.graphpad.com/, version 9.1.2, accessed on 2 June 2021).

## 3. Results

### 3.1. GJD2 mRNA Is Sparsely Expressed in HVC and Ubiquitously in RA

**Figure 1:**
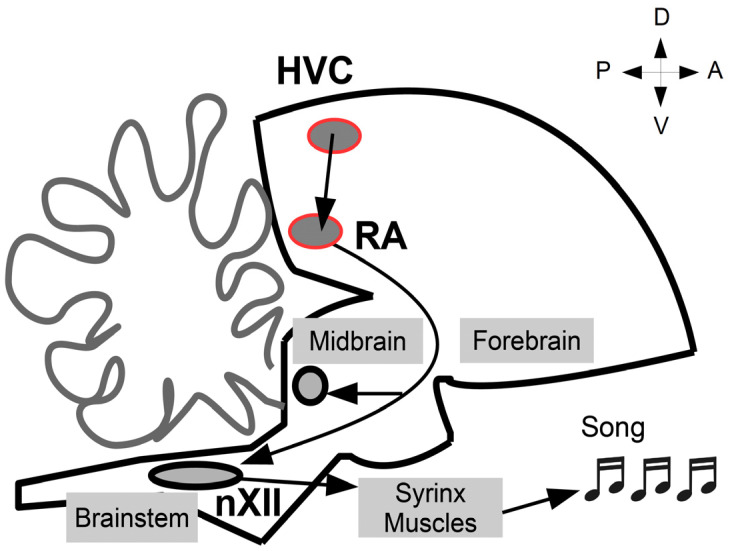
Song system motor pathway. The motor pathway controlling song production starts in the sensorimotor nucleus HVC (proper name). HVC synapses onto premotor nucleus RA (the robust nucleus of the arcopallium), the latter projecting onto brainstem motor nucleus nXII, which drives the contraction of the muscles of the vocal organ, the syrinx, producing songs. A, anterior; P, posterior; D, dorsal; V, ventral.

We investigated the expression of GJD2 in the two nuclei from the song premotor circuits, HVC and RA. At the apex of the motor pathway, nucleus HVC projects onto nucleus RA, which is only two synapses away from the vocal muscles (Figure 1).

**Figure 2:**
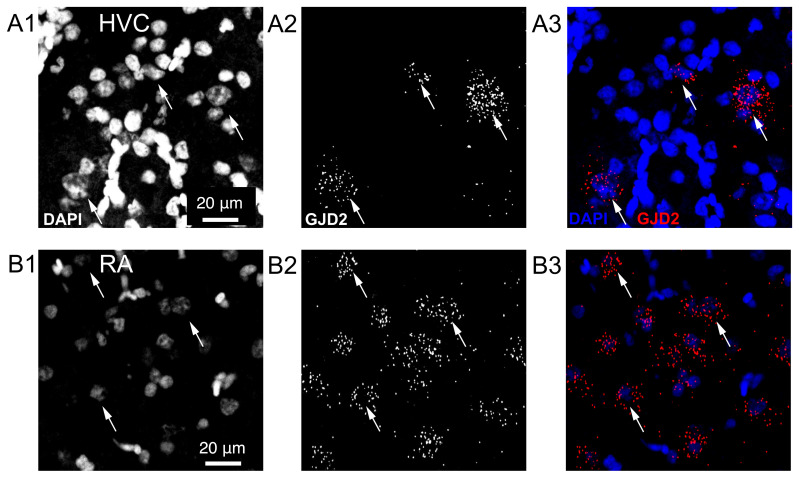
Expression of GJD2 mRNA in song nuclei HVC and RA from canaries. GJD2 mRNA labeling was counterstained by the nuclear staining, DAPI. (**A1**–**A3**) expression of GJD2 in a small fraction of cells in nucleus HVC. (**B1**–**B3**) widespread expression of GJD2 mRNA in nucleus RA. Single confocal planes. Arrows point toward three GJD2 mRNA-positive cells in each nucleus.

**Figure 3:**
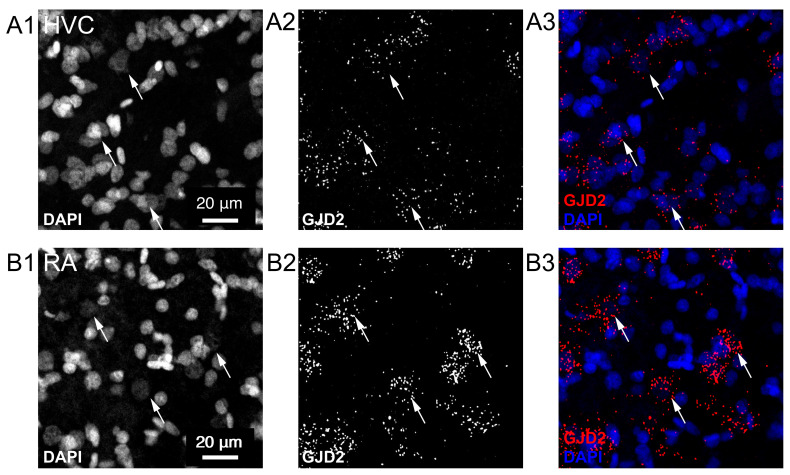
Expression of GJD2 mRNA in song nuclei HVC and RA from the zebra finch. GJD2 mRNA labeling was counterstained by the nuclear staining, DAPI. (**A1**–**A3**) expression of GJD2 mRNA in song nucleus HVC from zebra finches. (**B1**–**B3**) expression of GJD2 mRNA in song nucleus RA. Single confocal planes. Arrows point toward three GJD2 mRNA-positive cells in each nucleus.

We detected GJD2 mRNAs with fluorescent in situ hybridizations. In HVC, GJD2 mRNAs were sparsely found: only a small fraction of cells expressed GJD2 mRNA in adult male canaries (*Serinus canaria*, n = 9, Figure 2A1–A3). By contrast, in downstream nucleus RA, GJD2 mRNA was expressed in a large fraction of cells (Figure 2B1–B3). We confirmed that a similar expression pattern was found in males from another songbird species, zebra finches (*Taeniopygia guttata*, Figure 3).

### 3.2. GJD2 mRNA Expression in GABAergic HVC Neurons and A Subset of Glutamatergic HVC Neurons

**Figure 4:**
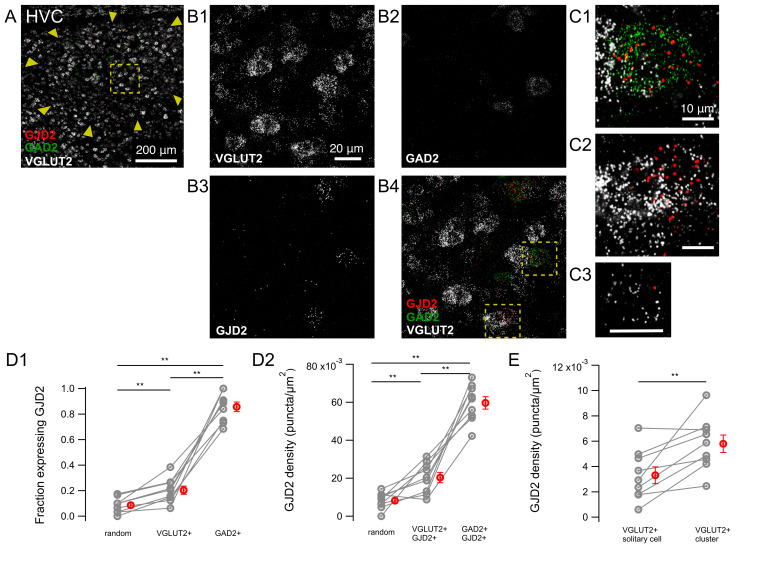
Expression of GJD2 mRNA in nucleus HVC from canaries. GJD2 mRNA is highly expressed in GAD2 mRNA-positive neurons and in only a fraction of excitatory cells coexpressing VGLUT2 mRNA. (**A**) HVC nucleus overview. (**B1**–**B4**) Magnification from the region highlighted in A. (**C1**,**C2**) insets show magnifications from regions highlighted in B4 of a GAD2 mRNA-positive, GJD2 mRNA-positive cell and a VGLUT2 mRNA-positive, GJD2 mRNA-positive cell cluster. (**C3**) GJD2 expression in a solitary excitatory cell. Scale bars in C1-C3, 10 μm. (**D1**) fraction of cells expressing GJD2 within each population of labeled neurons: VGLUT2 mRNA-positive and GAD2 mRNA-positive cells. ‘Random’, randomly drawn ROIs which did not contain DAPI staining and which were neither stained for DAPI nor for VGLUT2/GAD2. (**D2**) density of GJD2 mRNA puncta among VGLUT2 mRNA-positive and GAD2 mRNA-positive cells expressing GJD2 mRNA. (**E**) Comparison of the average GJD2 mRNA density in solitary cells and in cell clusters, defined as groups of cells formed by two or more adjacent cells. ** *P* < 0.01, two-tailed Wilcoxon signed-rank test. Each gray symbol represents the average for one animal. The average values of the same animal for the three types of ROI are linked together. Red symbols, average across animals. Error bars: ±S.E.M.

We examined the expression of GJD2 mRNA in excitatory and inhibitory cells by simultaneously performing in situ hybridizations targeting glutamatergic and GABAergic neurons. For that purpose, we used probes hybridizing with RNAs for the glutamate decarboxylase 2 enzyme (GAD2) found in GABAergic cells and probes hybridizing with RNAs for the vesicular glutamate transporter (VGLUT2) found in glutamatergic cells (Figure 4).

In HVC, the fraction of GJD2 mRNA-positive cells was 20.3 ± 3.1% among VGLUT2-mRNA-positive ‘solitary’ cells, i.e., cells that were not adjacent to other cells (n = 9 birds). This value was significantly lower than the fraction of GAD2 mRNA-positive cells that coexpressed GJD2 mRNA (85.7 ± 3.8%, n = 9 birds; two-tailed Wilcoxon signed-rank test, *P* = 0.004; Figure 4D1). The fraction of VGLUT2 mRNA-positive cells expressing GJD2 mRNA was significantly larger than the fraction of randomly placed ROIs which were neither stained for DAPI nor VGLUT2/GAD2, expressing GJD2 (8.6 ± 2.0%) (two-tailed Wilcoxon signed-rank test, *P* = 0.004), confirming that excitatory cells in HVC express GJD2 mRNA above background levels.

We further quantified on the same HVC confocal images the density of GJD2 transcripts among GJD2 mRNA-positive cells of both glutamatergic and GABAergic cell types. GAD2 mRNA-positive cells expressed a three times larger density of GJD2 transcripts (0.060 ± 0.003 puncta/μm^2^) than VGLUT2 mRNA-positive cells (0.020 ± 0.003 puncta/μm^2^, two-tailed Wilcoxon signed-rank test, *P* = 0.008; Figure 4D2) and the latter more than randomly placed ROIs (0.008 ± 0.001 puncta/μm^2^, two-tailed Wilcoxon signed-rank test, *P* = 0.004).

Since HVC neurons are often arranged in groups of several spatially clustered cells [24], we compared the expression level of GJD2 transcripts in excitatory cells that belonged to a cluster of cells (defined as a group of two or more adjacent cells) with solitary excitatory cells (Figure 4E). The average density of GJD2 mRNA in VGLUT2 mRNA-positive cells forming excitatory clusters was 0.0058 ± 0.0007 puncta/μm^2^, significantly larger than the density of 0.0033 ± 0.0007 puncta/μm^2^ found on average in solitary cells, including cells not expressing GJD2 mRNA (two-tailed Wilcoxon signed-rank test; *P* = 0.001).

Overall, in nucleus HVC, a larger fraction of cells expressing GJD2 mRNA and a higher density of GJD2 mRNA was found in inhibitory cells relative to excitatory cells, whose GJD2 expression level is higher in clusters than in solitary cells.

### 3.3. GJD2 mRNA Expression in RA GABAergic and Glutamatergic Neurons

**Figure 5:**
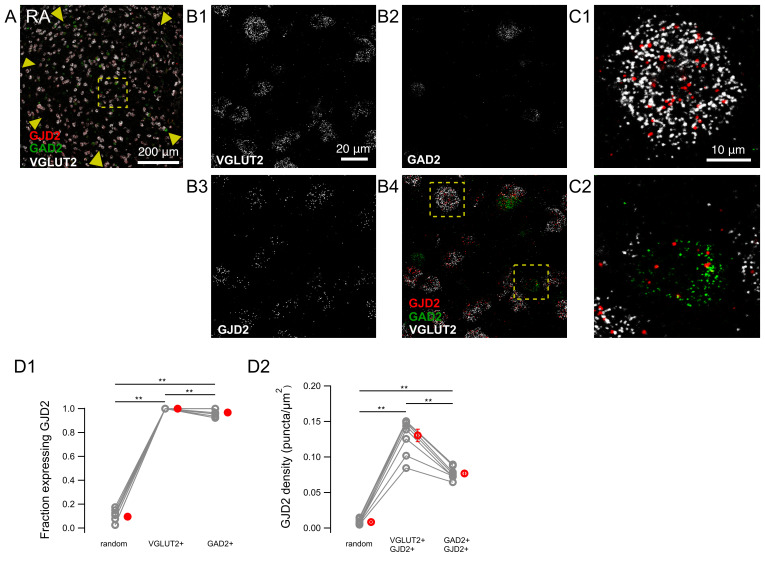
Expression of GJD2 mRNA in nucleus RA from canaries. Extensive expression of GJD2 in GAD2 mRNA-positive and VGLUT2 mRNA-positive neurons. (**A**) RA nucleus overview. (**B1**–**B4**) single confocal plane, magnification of the region highlighted in A. (**C1**–**C2**) Magnified insets from regions highlighted in B4 show a representative VGLUT2 mRNA-positive, GJD2 mRNA-positive cell and a representative GAD2 mRNA-positive, GJD2 mRNA-positive cell. (**D1**) fraction of cells expressing GJD2 within each population of labeled neurons: VGLUT2-positive and GAD2-positive cells. ‘Random’, randomly drawn ROIs which did not include DAPI staining and which were neither stained for DAPI nor for VGLUT2/GAD2. (**D2**) GJD2 density in GJD2 mRNA-expressing VGLUT2 and GAD2 mRNA-positive neurons. ** *P* < 0.01, two-tailed Wilcoxon signed-rank test. Each gray symbol represents the average for one animal. The average values of the same animal for the three types of ROI are linked. Red symbols: average across animals. Error bars: ±S.E.M.

We next quantified the expression of GJD2 mRNA in downstream nucleus RA (Figure 5). GJD2 mRNA was expressed in the large majority of GAD2 mRNA-positive and in all VGLUT2 mRNA-positive cells (95.2 ± 1.9% and 100.0 ± 0.0%, respectively, n = 8 birds; Figure 5D1).

GJD2 mRNA density was 0.130 ± 0.009 puncta/μm^2^ and 0.077 ± 0.003 puncta/μm^2^ in VGLUT2 mRNA-positive and GAD2 mRNA-positive cells expressing GJD2 mRNA, respectively, both significantly larger than control ‘random’ ROIs (0.009 ± 0.001; two-tailed Wilcoxon signed-rank test, *P* = 0.008; Figure 5D2).

A larger fraction of RA glutamatergic and GABAergic cells expressed GJD2 mRNA relative to the same cell types in HVC (Wilcoxon–Mann–Whitney two-sample rank test, *P* = 8 × 10^−5^ and *P* = 0.002, respectively). Likewise, when compared with nucleus HVC, GJD2 mRNA density of GJD2 mRNA-positive excitatory and inhibitory neurons was higher in RA than in HVC (two-tailed Wilcoxon–Mann–Whitney two-sample rank test, *P* = 8.2 × 10^−5^ and *P* = 0.002, respectively).

### 3.4. GJD2 mRNA Expression in the Nidopallium

We asked whether the cell-type identity of cells expressing GJD2 differed in both song nuclei, HVC and RA, relative to their expression in the regions these nuclei are embedded in. HVC and RA, respectively, develop from the nidopallium and the arcopallium, and have been proposed to evolve from these regions by circuit duplication [25].

**Figure 6:**
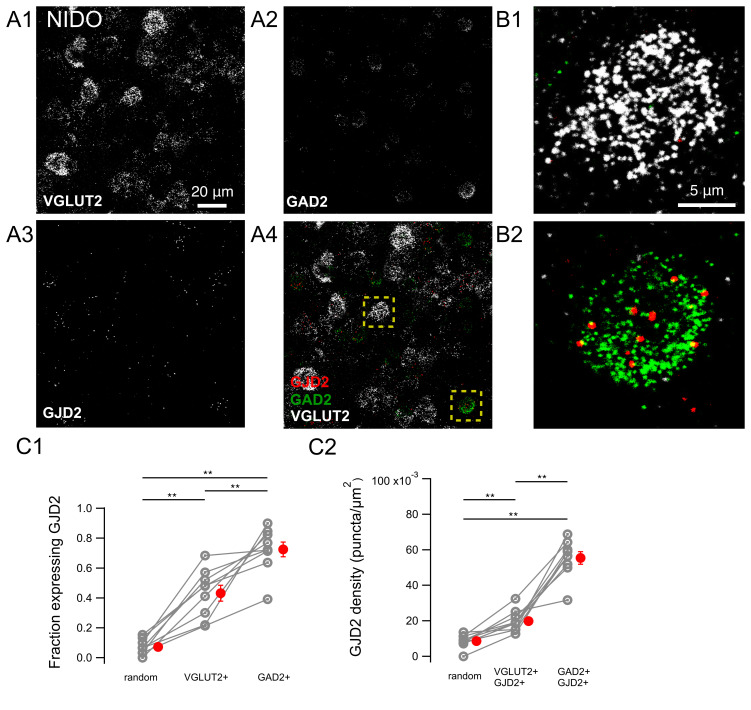
Expression of GJD2 mRNA in the nidopallium from canaries. GJD2 mRNA is expressed by a larger fraction of GAD2 mRNA-positive than VGLUT2 mRNA-positive neurons. (**A1**–**A4**) single confocal planes from the nidopallium. (**B1**,**B2**) magnification of representative VGLUT2 mRNA-positive and GAD2 mRNA-positive cells from regions highlighted in A4. (**C1**) fraction of cells expressing GJD2 within each population of labeled neurons: VGLUT2 mRNA-positive and GAD2 mRNA-positive cells. ‘Random’, randomly drawn ROIs which did not include DAPI staining and which were neither stained for DAPI nor for VGLUT2/GAD2. (**C2**) GJD2 mRNA density of GJD2 mRNA-expressing VGLUT2 and GAD2 mRNA-positive neurons. ** *P* < 0.01, two-tailed Wilcoxon signed-rank test. Each gray symbol represents the average for one animal. The average values of one animal for the three types of ROI are linked by a line. Red symbols, average across animals. Error bars: ±S.E.M.

For this purpose, we quantified GJD2 mRNA expression in the nidopallium in which HVC is embedded (Figure 6). GJD2 mRNA was expressed in 43.1 ± 5.4% of VGLUT2-positive cells and in 72.5 ± 4.9% of GAD2-positive cells (n = 9 birds; Figure 6C1). GJD2-positive cells from each cell type expressed 0.020 ± 0.002 puncta/μm^2^ and 0.055 ± 0.004 puncta/μm^2^, respectively (Figure 6C2).

The results are comparable to those found in HVC, where, similarly, a larger fraction of GAD2 mRNA-positive cells expressed GJD2 mRNA and at about three times higher expression levels than VGLUT2 mRNA-positive cells.

### 3.5. GJD2 mRNA Expression in the Arcopallium

**Figure 7:**
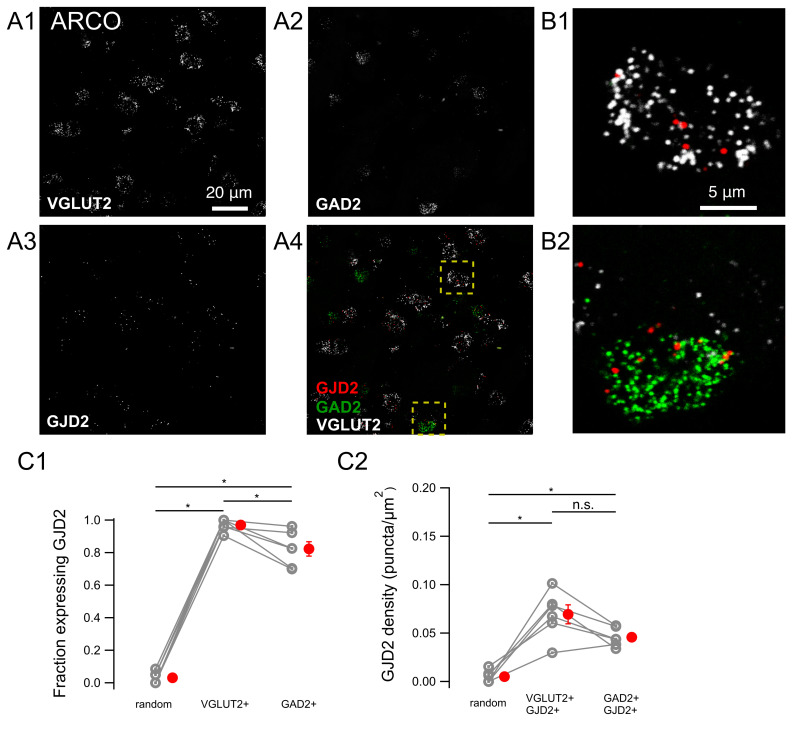
Expression of GJD2 mRNA in the arcopallium from canaries. GJD2 mRNA expression in the arcopallium in both GAD2 mRNA-positive and VGLUT2 mRNA-positive neurons. (**A1**–**A4**) single confocal planes. (**B1**–**B2**) magnification of representative VGLUT2 and GAD2 mRNA-positive cells from the regions highlighted in A4. (**C1**) fraction of cells expressing GJD2 within each population of labeled neurons: VGLUT2-positive and GAD2-positive cells. ‘Random’, expression in ROIs in the image which did not include DAPI staining and which were neither stained for DAPI nor for VGLUT2/GAD2. (**C2**) GJD2 mRNA density of GJD2 mRNA-expressing VGLUT2 and GAD2 mRNA-positive neurons. * *P* < 0.05; n.s., nonsignificant, two-tailed Wilcoxon signed-rank test. Each gray symbol represents the average for one animal. The average values of one animal for the three types of ROI are linked by a line. Red symbols: average across animals. Error bars: ±S.E.M.

We next quantified GJD2 mRNA expression in the arcopallium, the brain region in which RA is embedded. GJD2 mRNA was expressed in 97.0 ± 1.6% of VGLUT2 mRNA-positive cells and in 82.3 ± 4.4% of GAD2 mRNA-positive cells (n = 6 birds; Figure 7A–C). GJD2 mRNA-positive cells of each cell type expressed 0.069 ± 0.010 puncta/μm^2^ and 0.046 ± 0.004 puncta/μm^2^, respectively.

Thus, similarly to RA, GJD2 mRNA is strongly expressed in the large majority of VGLUT2 and GAD2 mRNA-positive cells, the fraction of expressing cells and the level of expression being higher in VGLUT2 mRNA-positive cells.

### 3.6. GJD2 Expression in Single-Cell RNA Sequencing Data

**Figure 8:**
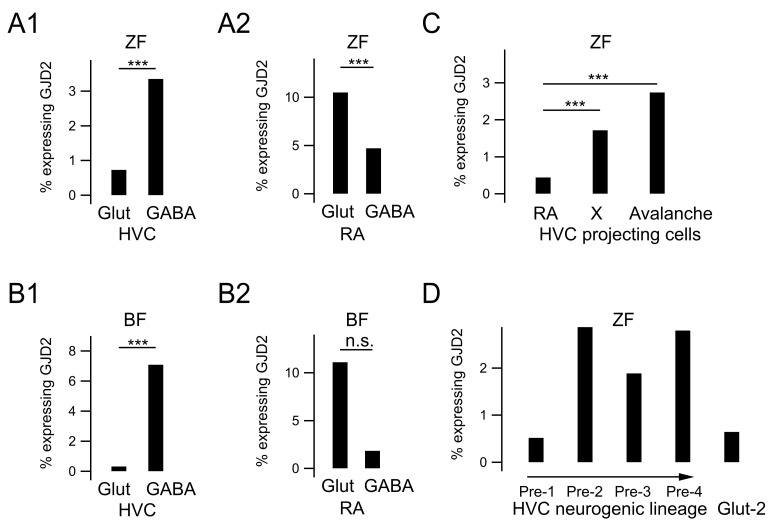
Fraction of GJD2-expressing neurons from single-cell transcriptomics data. Fraction of GJD2-expressing neurons in A. zebra finches (ZF) and B. Bengalese finches (BF). (**A1**,**B1**) Percentage of excitatory and inhibitory cells expressing GJD2 in HVC. (**A2**,**B2**) Percentage of excitatory and inhibitory cells expressing GJD2 in RA. (**C**) Percentage of cells expressing GJD2 in different HVC glutamatergic projection neuronal subtypes. (**D**) Percentage of cells expressing GJD2 in clusters identified as belonging to the neurogenic lineage (Pre-1 to Pre-4) and immature neurons (cluster Glut-2). The arrow indicates the progression of cells along the neurogenic lineage, from stem cells (Pre-1) to late stages (Pre-4). *** *P* < 0.001; n.s., nonsignificant, Fisher’s exact test.

Finally, we compared our results with recently published single-cell sequencing data from two songbird species: zebra finches and Bengalese finches [26] (https://cloud.biohpc.swmed.edu/index.php/s/nLicEtkmjGGmRF8?path=%2F accessed on 2 July 2021). In both species, a larger fraction of HVC GABAergic neurons (3.35% and 7.09% for zebra and Bengalese finches, respectively) expressed GJD2 mRNA than glutamatergic neurons (0.73% and 0.32% for zebra and Bengalese finches, respectively, Fisher’s exact test, *P* < 0.0001; Figure 8A1,B1), similarly to our results in canaries. Likewise, a larger fraction of RA glutamatergic cells (10.50%) expressed GJD2 mRNA than RA GABAergic cells in zebra finches (4.72%, Fisher’s exact test, *P* < 0.0001), and a similar, although a nonsignificant trend was found in Bengalese finches (11.11% vs. 1.45%, Fisher’s exact test, *P* = 0.0974, Figure 8A2,B2). Finally, the dataset shows a stronger expression of GJD2 in RA than in HVC, confirming the results of our in situ hybridizations (Figure 8). Note that the fractions of cells expressing GJD2 detected in our dataset were one order of magnitude larger than those extracted from single-cell sequencing data [26] presented in this section, likely due to the higher sensitivity of in situ hybridizations as will be further developed in the discussion.

We next asked whether all subtypes of glutamatergic cells in HVC from the same dataset expressed GJD2 and whether GJD2 expression differed among glutamatergic cellular subtypes. Cell types identified by [26] as putatively belonging to populations of HVC cells projecting to RA, X and Avalanche, as well as immature cells, all contain cells expressing GJD2 mRNA. Different glutamatergic cell subtypes presented different fractions of GJD2 mRNA-positive cells (Figure 8C). In zebra finches, the species for which the dataset was large enough to perform comparisons among glutamatergic cell types, a larger fraction of HVC cells projecting to X (1.72%) and to Avalanche (2.74%) expressed GJD2 mRNA relative to neurons projecting to RA (0.44%; Fisher’s exact test, *P* < 0.0001 and *P* = 0.0009, respectively) and immature neurons (HVC_Glut-2 cluster, 0.64%, *P* = 0.0035 and *P* = 0.0067, respectively).

Finally, putative adult neural stem cells (cluster HVC_Pre-1) generate cells that differentiate into neurons and comprise intermediate neural precursors or migrating neuroblasts (clusters HVC_Pre-2 and HVC_Pre-3) and more differentiated cells, closer-to-mature neurons (cluster HVC_Pre-4) [26]. These three subtypes of cells differentiating into a neuronal phenotype expressed GJD2 (2.87%, 1.89% and 2.80% of respective populations). Interestingly, although the fraction of stem cells (HVC_Pre-1 cluster) expressing GJD2 was low (0.52%), this fraction increased in intermediate differentiation steps (HVC_Pre-2 to HVC_Pre-4, *P* = 0.0004) and decreased again at the terminus of the neurogenic lineage (HVC_Glut-2, 0.64%, *P* < 0.0001, Figure 8D).

In summary, single-cell transcriptomics datasets from zebra and Bengalese finches confirm and generalize to two additional songbird species the distributions of electrical synapses across cell types and song nuclei that we found in HVC and RA from canaries. Furthermore, they reveal GJD2 expression in specific glutamatergic subtypes: a larger fraction of HVC cells projecting to nuclei X and Avalanche expressed GJD2 mRNA relative to immature neurons and neurons projecting to RA. Moreover, datasets allow investigating the transient increase in GJD2 expression in differentiating neurons as neural precursors differentiate into neurons along the neurogenic pathway.

## 4. Discussion

GJD2 mRNA is extensively expressed in the song motor pathway of three songbird species, producing very different songs: canaries, zebra finches and Bengalese finches. These songbird species differ in their song properties, song learning strategies and neural plasticity. Canaries produce long tours of frequency-modulated song syllables with variable syllable syntax, whereas zebra and Bengalese finches produce short stacks of predominantly harmonic notes and a smaller syllable repertoire. Bengalese finches display variable syntax in comparison to the fixed sequencing of zebra finch songs. The GJD2 expression throughout the year and across songbird species that sing different songs suggests that electrical synapses and their distributions across cell types and song nuclei are basic features of song premotor neuronal networks.

Our in situ hybridizations show that both song control nuclei, HVC and RA, express the gene GJD2 in excitatory and inhibitory neuronal populations in canaries, with different expression profiles. Whereas in HVC, most GABAergic cells and only a fraction of HVC glutamatergic cells express GJD2 mRNA, both cell types extensively express GJD2 in RA, with a stronger expression in glutamatergic cells. Interestingly, similar relative expression patterns in GABAergic vs. glutamatergic cells can also be found in recently published single-cell transcriptomics data for zebra and Bengalese finches [26]. Moreover, the dataset reveals a transient increase in GJD2 mRNA expression by differentiating neurons before they reach the final stages of neuronal differentiation.

The expression of GJD2 mRNA in HVC and RA and the respective regions in which they are embedded reveals similar cell-type-specific expression patterns, thus suggesting no major evolutionary pressure to change the electrical synaptic phenotype of cell types in song motor nuclei relative to their regions of origin.

Here, we provide a quantitative characterization, for the first time, of GJD2 expression in neurons from the avian brain. The ubiquitous expression of electrical synapses formed by connexin 36 has so far been extensively documented in both fish and mammals, in which it is encoded by orthologous genes [27]. Thus, the present study extends our knowledge on the expression of electrical synapses formed by connexin 36 in the vertebrate brain to the avian brain, where we demonstrate that GJD2 is expressed at previously unsuspected levels.

### 4.1. GJD2 in the Song Control Circuits

Our current understanding and models of neural circuits in the song system only include chemical synapses [28] but lack electrical synapses, which we demonstrate here to be strongly expressed in song nuclei. Given the strong impact of electrical synapses on the firing dynamics and computations performed by neural networks [3], including electrical synapses in models of HVC and RA is expected to strongly impact the generation of network firing dynamics and coding in the song system. Specifically, the contribution of electrical synapses to neuronal properties, network activation and singing behavior in the different cell types and nuclei are three open directions that should be further pursued.

Based on the known impact of electrical synapses on the action potential firing dynamics of coupled networks [1,2,3,4,5,6,7], we hypothesize that electrical synapses may contribute to the generation of synchronous and sequential activity of cell ensembles in both HVC and RA and thereby to song temporal and spectral features. Electrical synapses could, moreover, increase the selectivity of neuronal firing in response to specific input patterns onto both coupled networks while decreasing it in response to other patterns [7]. However, predicting the function of electrical synapses in HVC and RA is at this point difficult because of both the variable impact of electrical synapses on neural networks [2] and the need to characterize the connectivity of specific cell types via electrical synapses in both nuclei.

The high expression level of GJD2 mRNA in HVC interneurons suggests that interneurons are interconnected via electrical synapses. Moreover, a fraction of excitatory cells also express GJD2 mRNA in HVC. Some of the glutamatergic cells positive for GJD2 mRNA are likely responsible for the gap junctions described between somata in HVC clusters [29]. In RA, the high expression of GJD2 mRNA in both cell types leaves open the different combinations of putatively connected cell types. Future work should determine the connectivity patterns in both nuclei via electrical synapses. Since excitatory and inhibitory cells form heterogeneous populations both in HVC and RA [26], the cell-type-specific network connectivity via electrical synapses should be characterized among the different glutamatergic and GABAergic cell subtypes.

The expression of GJD2 in HVC is in agreement with previously described dye coupling in HVC [30] and somatic gap junctions among HVC cells in clusters [29]. Here, we found that not only cell clusters formed by excitatory cells but also solitary glutamatergic cells and GABAergic interneurons express GJD2. Thus, we predict that electrical synapses are also located in neurites from both excitatory and inhibitory cells in both song nuclei.

Interestingly, the expression of GJD2 increases during neuronal differentiation and decreases as progenitors mature. These changes in the GJD2 expression pattern along the neurogenic lineage suggest that electrical synapses transiently increase coupling once adult-born neurons enter intermediate differentiating stages, decreasing GJD2 expression upon final maturation of circuits. Thus, GJD2 may play a role in the migration, maturation and integration of adult-born differentiating neurons. Since the integration of adult-born neurons has been proposed to contribute to song learning [31], the transient increase in GJD2 expression may contribute to the functional integration of newborn cells and thereby, to learning. Remarkably, this maturation pattern reminds the well-established increase in electrical coupling during the maturation of neural circuits and its decrease in mature circuits described in mammalian brains, particularly in the neocortex [32,33].

### 4.2. Comparison of in Situ Hybridizations and Single-Cell Transcriptomics

Transcriptomic data confirmed the expression of GJD2 in glutamatergic and GABAergic cells in HVC and RA from zebra and Bengalese finches that we found with in situ hybridizations in canaries. In situ hybridizations have an excellent sensitivity and signal-to-noise ratio, therefore making it possible to detect low amounts of transcripts and perform a thorough quantitative analysis of GJD2 mRNA expression. In comparison, single-cell sequencing data may have a high false-negative rate in detecting the presence of GJD2 due to the ‘dropout’ property of the method. This was apparent when we compared our in situ hybridization results with single-cell transcriptomics data. Whereas 100% of glutamatergic cells expressed GJD2 mRNA in RA as detected with in situ hybridizations, only ~10% of glutamatergic cells were positive for GJD2 with single-cell transcriptomics [26]. The difference between the two methods was of one order of magnitude in the fraction of GJD2+ cells, but the cell type specificity of expression was consistent. This difference of one order of magnitude in the fraction of cells positive for GJD2 is likely intrinsically due to the limiting level of detection of some scRNA-seq methods and sequencing depth, which can miss the detection of low-level expression genes [34]. Recently, however, [35] successfully detected a robust expression of GJD2 in mammalian cerebellar molecular layer interneurons by scRNA-seq, proposing GJD2 as a marker for an interneuron subtype.

Overall, single-cell transcriptomics proved highly valuable to confirm the cell-type specificity of GJD2 expression in two additional species and identify GJD2 expression in cellular subtypes, which were characterized by clustering based on gene expression patterns. Single-cell transcriptomics made it possible to compare GJD2 expression in different HVC glutamatergic projection cells and newborn cells, revealing that cells projecting to X and Avalanche show a larger expression than cells projecting to RA and allowing to track GJD2 expression dynamics along the neurogenic lineage, characterized by a transient increase in GJD2 expression as neurons differentiate.

### 4.3. Electrical Synaptic Phenotype of Song Nuclei: Evolutionary Considerations

Both glutamatergic and GABAergic cells within the two song control nuclei HVC and RA express GJD2. Similarly, GABAergic and glutamatergic neurons of the tissues that embed HVC and RA, respectively, express GJD2 with similar cell-type-specific expression patterns. Thus, it is likely that the electrical synaptic phenotype of cells was acquired in primitive nidopallial and arcopallial networks before the specialization of song motor nuclei. Note that for many other genes, on the contrary, song nucleus-specific gene expression has been documented (http://www.zebrafinchatlas.org/ accessed on 21 August 2021; [36,37,38]). Our results, therefore, suggest that there has been no major evolutionary pressure to modify the cell-type expression pattern of the gene GJD2 in song control nuclei HVC and RA relative to the expression in the regions they evolved from. Thus, electrical synapses may form a constitutive element of arcopallial and nidopallial networks.

Finally, since canaries, zebra and Bengalese finches are members of different songbird families (Fringillidae and Estrildidae) [39] the expression of connexin 36 in the song control system and its distribution across cell types and song nuclei are likely a general feature of songbirds. As the neuronal control of birdsong shows both a large developmental and adult plasticity, the investigation of electrical synapses and their plasticity in songbirds is likely to advance our understanding of the synaptic basis of learned complex behaviors.

## 5. Conclusions

GJD2 is extensively expressed in neurons from the song motor pathway. In song nucleus HVC, GJD2 is widely expressed in GABAergic cells and in only a fraction of glutamatergic cells at lower expression levels and differentially among glutamatergic cells subtypes. Within the neurogenic lineage, GJD2 mRNA expression transiently increases as neurons differentiate. GJD2 mRNA expression is higher in RA than in HVC, with an extensive expression in both RA GABAergic and glutamatergic cell types, higher in the latter. Overall, the widespread expression of GJD2 in song premotor networks suggests that electrical synapses may play a major role in coordinating the activity of neural networks responsible for song production. Moreover, expression of GJD2 mRNA across cell types is similar in HVC and its surrounding region, the nidopallium, and in RA and its surrounding region, the arcopallium, suggesting no evolutionary pressure to modify the cell-type-specific GJD2 expression in song control nuclei HVC and RA, and revealing the widespread expression of GJD2 in the avian brain.

## Data Availability

The datasets analyzed in the current study are available from the corresponding author on request.

## Abbreviations

RA	the robust nucleus of the arcopallium
HVC	previously used as an abbreviation of ‘High Vocal Center’; used here as a proper name
GJD2	gap junction protein delta 2
ROI	region of interest
VGLUT2	vesicular glutamate transporter 2
GAD2	glutamate decarboxylase 2
